# A model of faulty and faultless disagreement for post-hoc assessments of knowledge utilization in evidence-based policymaking

**DOI:** 10.1038/s41598-024-69012-3

**Published:** 2024-08-09

**Authors:** Remco Heesen, Hannah Rubin, Mike D. Schneider, Katie Woolaston, Alejandro Bortolus, Emelda E. Chukwu, Ricardo Kaufer, Veli Mitova, Anne Schwenkenbecher, Evangelina Schwindt, Helena Slanickova, Temitope O. Sogbanmu, Chad L. Hewitt

**Affiliations:** 1 Department of Philosophy, Logic and Scientific Method, London School of Economics and Political Science, London, UK; 2 Department of Philosophy, University of Missouri, Columbia, USA; 3 School of Law, Queensland University of Technology, Brisbane, Australia; 4 Instituto Patagónico Para El Estudio de los Ecosistemas Continentales (IPEEC-CONICET), Puerto Madryn, Argentina; 5 Center for Infectious Diseases Research, Nigerian Institute of Medical Research, Lagos, Nigeria; 6 Center for Interdisciplinary Research (ZiF), Bielefeld University, Bielefeld, Germany; 7 African Centre for Epistemology and Philosophy of Science, University of Johannesburg, Johannesburg, South Africa; 8 School of Humanities, Arts and Social Sciences, Murdoch University, Perth, Australia; 9 Instituto de Biología de Organismos Marinos (IBIOMAR-CONICET), Puerto Madryn, Argentina; 10 Faculty of Philosophy, University of Groningen, Groningen, the Netherlands; 11 Department of Zoology, University of Lagos, Lagos, Nigeria; 12 Environmental Evidence Synthesis and Knowledge Translation (EESKT) Research Group, University of Lagos, Lagos, Nigeria; 13 Centre for Biosecurity Research Analysis and Synthesis, Lincoln University, Lincoln, New Zealand; 14 Centre for Biosecurity and One Health, Murdoch University, Perth, Australia; 15 Academy for Territorial Development (ARL), Leibniz Association, Hannover, Germany

**Keywords:** Evidence-based policy, Disagreement, Transparency, Epistemology, Applied mathematics, Climate-change policy, Psychology and behaviour

## Abstract

When evidence-based policymaking is so often mired in disagreement and controversy, how can we know if the process is meeting its stated goals? We develop a novel mathematical model to study disagreements about adequate knowledge utilization, like those regarding wild horse culling, shark drumlines and facemask policies during pandemics. We find that, when stakeholders disagree, it is frequently impossible to tell whether any party is at fault. We demonstrate the need for a distinctive kind of transparency in evidence-based policymaking, which we call transparency of reasoning. Such transparency is critical to the success of the evidence-based policy movement, as without it, we will be unable to tell whether in any instance a policy was in fact based on evidence.

## Introduction

Disagreements over what conclusions can be drawn from a diverse body of evidence are a central feature of social life. Such disagreements take on particular importance within scientific research because disagreement and debate are central to scientific development ^1–3^. Disagreements concerning scientific research are further complicated by the relationships formed within the process of evidence-based policymaking (EBPM).

EBPM is an increasingly widespread approach used to base policy decisions on rigorously established information and purporting to protect the policymaking process from ideology, bias, prejudice, and other contingent factors. This goal is not simple to achieve. Evidence rarely speaks for itself, policies have multiple criteria by which they may succeed or fail, various parties value policy outcomes differently, bias and ideology can creep in, and so on ^4–7^. Amidst this inherent complexity, allegations of policy failure are frequently articulated in terms of disagreement about adequate knowledge utilization. For example, stakeholders often argue that policy on the management of wild horses is inconsistent with evidence on their ecological impact ^8,9^, lament that evidence on the ineffectiveness of lethal shark drumlines is ignored ^10,11^, or disagree with the evidence base for international policy on the use of facemasks during a pandemic ^12,13^. But there is substantial difficulty in objectively evaluating whether a purportedly evidence-based policy is consistent with adequate knowledge utilization ^14^. Where parties disagree over whether a suitable policy decision has been made given the available evidence, how can we tell what, if anything, went wrong with the use of that evidence through the policymaking process?

To help answer this question, we analyze a highly simplified and idealized model of knowledge utilization in decision-making, which singles out a basic feature of disagreement regarding evidence use in EBPM. This basic feature will infect any and all instances of EBPM, beyond the simple scenarios we describe. We demonstrate that faultless disagreement, i.e., disagreement not arising from any error in knowledge utilization such as misinterpretation or misapplication of evidence, can arise due to different methods of weighting evidence ^15,16^. Philosophers have variously referred to this as ‘reasonable disagreement﻿﻿’ or ‘﻿peer disagreement﻿’ ^17^. More generally, philosophers have discussed situations where things look like they have gone wrong even when no individual has done anything wrong. For instance, the ‘independence thesis﻿’ states that rational individuals might not make for rational groups and rational groups might not be composed of rational individuals ^18^, polarization can emerge in groups of perfectly rational agents ^19–21^, and permissible or justifiable differences in values can lead to differences in judgment ^22,23^. Similarly, we argue, faultless disagreement is possible in EBPM.

As such, a policy can be the result of adequate knowledge utilization in EBPM, even with disagreement in post-hoc assessments of whether the policy is suitably evidence-based. We also demonstrate that a major roadblock for the implementation of EBPM is the inability to discern whether a disagreement about policy is with or without fault, which is critical for evaluating post-hoc any avowed commitments to EBPM by relevant parties, as well as for building, maintaining, or repairing trust ^24^. This roadblock will be a foundational issue for case-based evaluations of EBPM even in the presence of various complications that we set aside here, like lobbying, political factors, and so on. EBPM thus requires a distinctive kind of transparency, which we call *transparency of reasoning*.

## Methods

We develop a simple, highly idealized mathematical model. To a reader familiar with the existing literature on EBPM that typically emphasizes inherent complexities involved, we acknowledge upfront that the model may appear lacking in nuance. But one should not confuse the model itself with the methodology that makes use of it, which complements existing approaches. In developing the simple model, our aim is to isolate a few points about understanding and evaluating disagreements in EBPM that are otherwise generally neglected in discussions of EBPM. We regard that neglect as a consequence of other methods that are more dominant in existing work on the topic, which favor faithfully preserving complexity. We believe that, since our model’s basic building blocks should also be present in more nuanced analyses of EBPM, our conclusions carry over to such analyses. But in those more nuanced analyses, those conclusions lurk deep beneath the surface. The simple model brings them out.

How so? The basic building blocks just mentioned are factors we take to be present in any attempt at EBPM: evidence, the weights parties put on it, and potential misinterpretation. In focusing on these factors, we deliberately set aside many complexities that a more ambitious, wholesale study of EBPM would have to take into account. In order to focus our attention on post-hoc disagreements over the extent to which evidence supports a particular policy proposal, for example, we assume agreement among all parties represented in the model about what the policy proposal amounts to, the kinds of evidence that are potentially relevant, whether particular data speaks for or against the policy proposal, and so on. We assume, effectively, that the complex processes of policy formulation and knowledge co-production, that might bring parties involved in EBPM to a shared understanding of what ‘evidence’ even means in a given policymaking context^25–27^, have already taken place. Other notable factors we ignore include outside actors influencing the policy process, interactions between local and national government bodies, and weighting multiple policies against each other.

The resulting model, in leaving out all these complexities, may look like a caricature of any real-life attempt at EBPM. The advantage of such a mathematical modeling approach, however, is that it makes clear what idealizations or abstractions are being made that ultimately explain the results we obtain. Since we use the model only to identify possible sources of disagreement, without ruling out other possibilities or making claims about how frequently particular kinds of disagreement occur, our conclusions about the nature of disagreement in EBPM apply more broadly to any real, more complex situation where the minimal elements of our model are present.

In the context of mathematical modeling, one otherwise ambiguous feature of disagreement in EBPM becomes immediately pressing: whether it is transient, as a function of what scientists and policymakers have learned about the world by any given point within the evidence-gathering process, or whether it reflects some difference between scientist and policymaker that can never be eliminated — perhaps due to the different positions they occupy within EBPM. Here, we primarily report on results of a statistical learning model that reflects the clear emergence of ineliminable disagreement between individuals ‘in the long run’ (as what they each claim to know about the underlying science grows vast). Hence, the results of this model highlight non-transient disagreement. This is not to deny that there are interesting questions about the effects of ineliminable disagreements already ‘in the medium run’, but to acknowledge a limitation of our chosen method. Below, we supplement our mathematical modeling with some computational modeling, to indicate at least one aspect of learning in EBPM which requires paying attention to the medium run.

In our model, there are two agents: individuals or groups whose beliefs we will track, who stand in for relevant actors broadly involved in EBPM (including scientists largely disengaged from any actual policymaking processes, who may nonetheless pass judgements on the policy uptake of the science). These agents receive evidence from two evidence streams (e.g., one on the ecological impact of wild horses and one on the value of wild horses to the public), both of which bear on a single binary policy decision (e.g., whether to cull the horses). We focus on a binary policy decision for clarity and simplicity, while acknowledging that real-life policy decisions typically involve more than two possible courses of action and/or a decision better described on a graded scale. We think all of the lessons we draw from our model carry over to such scenarios. Note also that we leave open the nature of evidence streams (see further discussion below) and, importantly, how it is decided which evidence streams to consider in the first place.

In what we will refer to as the ‘pristine case’ of the model, the two agents interpret the evidence from the two streams in the same way (e.g., they agree that ecological evidence suggests the horses are damaging the native ecosystem and that there are positive feelings towards the presence of the horses). In the pristine case, the only difference between the two agents is that they differ in how they weight the relevance of the evidence streams to the policy decision. This difference in weighting is at least potentially due to legitimate reasons (e.g., due to different value judgments or social roles, see the next section for more discussion). We study the highly idealized pristine case to highlight the possibility of faultless disagreement in the context of EBPM, defined as disagreement despite adequate knowledge utilization by all parties.

**Figure 1:**
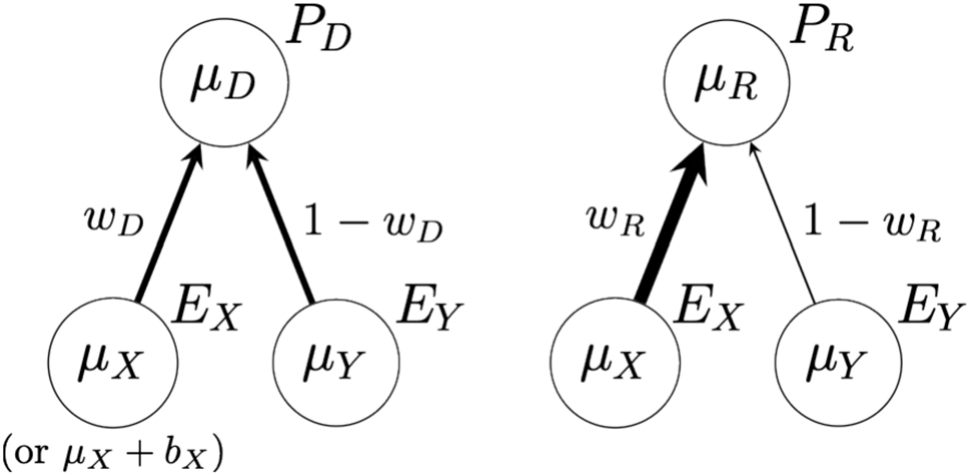
Two Evidence Streams, One Policy Decision. *P*_*D*_ and *P*_*R*_ reflect the evidential support for the binary policy decision at hand, as considered by agents *D* and *R*, respectively. Two evidence streams *E*_*X*_ and *E*_*Y*_ each bear on the policy decision. The agents receive data from the streams that lets them learn the true values of underlying parameter values (*μ*_*X*_ and *μ*_*Y*_, respectively). *μ*_*D*_ and *μ*_*R*_ are the (posterior) mean of each agent’s beliefs about the evidential support for the policy; they recommend in favor of the policy if this is above a threshold and against if it is below the threshold. In the pristine case, both agents have unbiased access to the evidence (in the non-pristine case, *D* sees biased evidence from stream *E*_*X*_ so her learning converges to *μ*_*X*_ + *b*_*X*_ instead). The agents judge the relevance of the two streams differently, as reflected in the weights on the arrows. In the limit of accumulated evidence, *μ*_*D*_ converges to *w*_*D*_*μ*_*X*_ + (1—*w*_*D*_)*μ*_*Y*_ and *μ*_*R*_ converges to *w*_*R*_*μ*_*X*_ + (1—*w*_*R*_)*μ*_*Y*_. If these quantities are different, it will produce support disagreement (see Fig. 2) and if they are sufficiently different to be on different sides of the threshold, it will produce policy disagreement with increasing probability as evidence accumulates (*Result 1*).

In the non-pristine case of our (still highly idealized) model, one agent learns one evidence stream in a faulty manner (interpreted as inadequate knowledge utilization), represented in the model as a systematic bias for or against the policy decision. The non-pristine case introduces a source of faulty disagreement. The primary aim of studying this case is to see what is required for disagreeing agents to be able to tell whether they are in a faulty or faultless disagreement. The basic elements of the model are summarized in Fig. 1.

**Figure 2:**
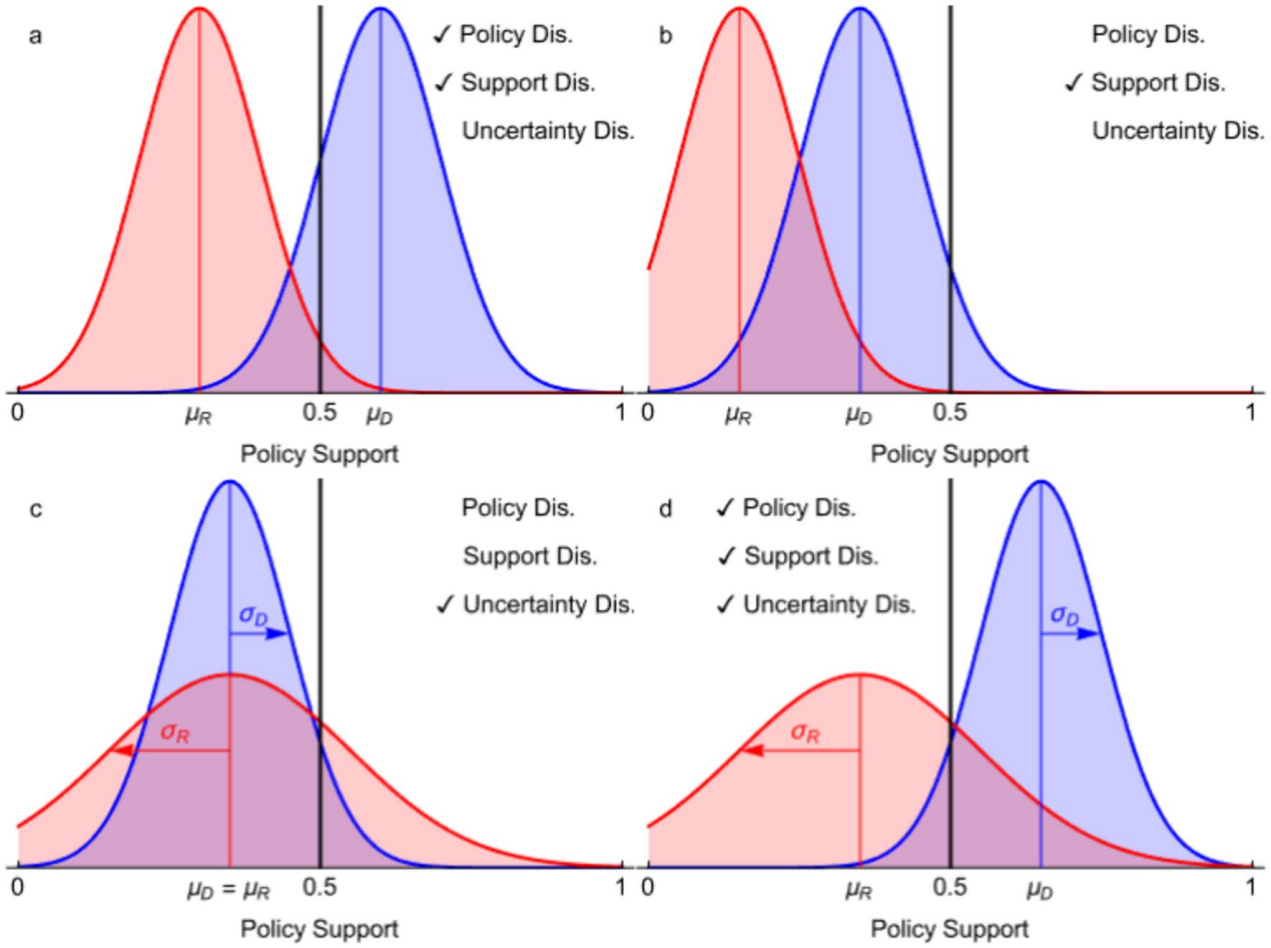
Three Kinds of Disagreement. Two agents’ (*D*—blue and *R*—red) posterior beliefs about the evidential support *P*_*D*_,* P*_*R*_ ∈ [0,1] for a binary policy decision (‘policy support’) after seeing evidence from two streams *E*_*X*_ and *E*_*Y*_. An agent recommends the policy if the mean of her belief (*μ*_*D*_ or *μ*_*R*_, respectively, see Fig. 1) exceeds a threshold, here one-half, otherwise she recommends against it. Policy disagreement occurs when the agents give different recommendations (subfigures **a**, **d**). Support disagreement occurs when the agents believe the policy support to be different (*μ*_*D*_ ≠ *μ*_*R*_), e.g., one thinks the evidence speaks very strongly against the policy, while the other is more ambivalent (**a**, **b**, **d**). Policy disagreement entails support disagreement but not vice versa. Uncertainty disagreement occurs when agents differ in how confident they are in their judgment of policy support, as measured in the standard deviation (*σ*_*D*_ or *σ*_*R*_, respectively) of their posterior beliefs (**c**, **d**).

Even in our simple model, at least three different kinds of disagreement are possible. *Support disagreement* occurs when two agents disagree about the level of support the evidence provides for a particular binary policy decision. *Policy disagreement* is a special kind of support disagreement where the disagreement leads one agent to recommend in favor of the policy decision and the other against. An example of policy disagreement is when ecologists and policymakers disagree on whether a wild horse culling policy should be implemented. *Uncertainty disagreement* occurs when the agents have different degrees of confidence in their recommendation. The three kinds of disagreement are illustrated in Fig. 2.

Finally, we briefly study computationally the effects of variable learning speeds across different evidence streams, as they affect learning in the medium run. Our analytic results focus on what happens in the model in the large sample limit. For such results, it does not matter how quickly the data from the two evidence streams comes in, relative to each other. Simulation lets us highlight what happens in the model before enough evidence is seen that limiting behavior dominates the analysis, without taking away from the main conclusions of the large sample analysis.

## Results

**Figure 3:**
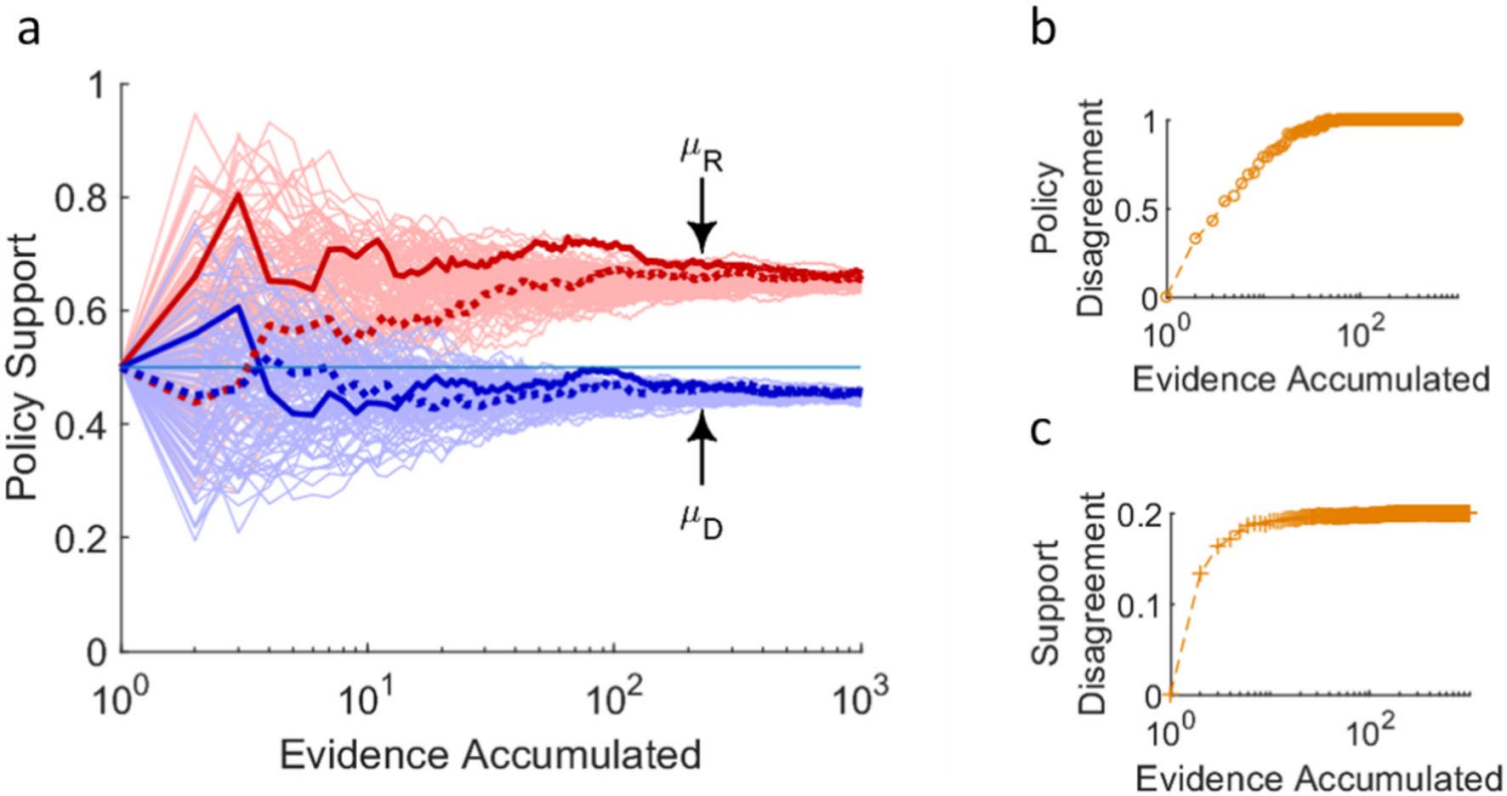
Policy Disagreement Illustrated with Simulation Data. This figure shows a hundred paired simulation runs in which two agents (*D*—blue and *R*—red) see Gaussian data from two evidence streams (*μ*_*X*_ = 0.7 and *μ*_*Y*_ = 0.2 are unknown to the agents, *σ*_*X*_ = *σ*_*Y*_ = 0.25 known), which they weight differently (*w*_*D*_ = 0.5, *w*_*R*_ = 0.9). On the *x*-axis, evidence accumulates as measured by the number of Gaussian data points from each stream agents have seen (on a log scale). As the evidence comes in, the agents’ beliefs about the evidential support for the policy evolve from a prior (*m* = 0.5), settling near 0.45 for *D* and 0.65 for *R*. We highlight two simulation runs (bold and dashed lines in subfigure **a**) to illustrate that the two agents’ trajectories are correlated because they see the same data. We measure policy disagreement as the proportion of simulation runs in which the agents’ recommendations differ; here, all one hundred runs end in policy disagreement (*R* recommends in favor of the policy, *D* against) once agents’ beliefs settle (**b**). We measure support disagreement as the distance between *μ*_*D*_ and *μ*_*R*_; here, this settles near 0.2 (**c**). See Data S1 for simulation code.

Our first result (*Result 1*, Supplementary Materials) is that there can be policy disagreement and support disagreement in the pristine case of the model. That is, agents can come to disagree about whether and to what extent a policy is a good idea resulting solely from the different weights they each put on the evidence streams. This is not necessarily a surprising result, at least once the question has been put forward, but it shows that the model does what we expect a model for studying disagreements to do. Moreover, the model lets us talk about the kinds of disagreement and the assumptions required to obtain them with greater precision than if we were reasoning informally (see Fig. 3 and the Supplementary Materials).

**Figure 4:**
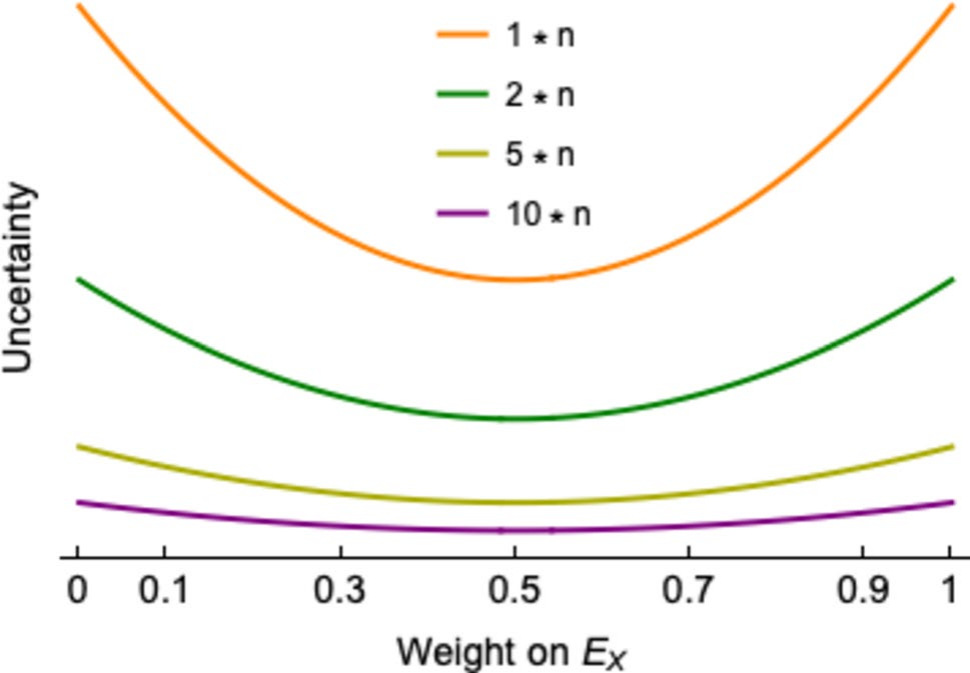
Uncertainty Disagreement in the Pristine Case. In the pristine case of the model, the only difference between the agents is in the weights they put on the two evidence streams. The figure shows that whichever agent is closer to weighting the streams equally will be less uncertain in her beliefs about evidential support for the policy (compared to another agent with the same amount of evidence but more extreme weights), as measured in the posterior variance (*σ*_*D*_^2^ or *σ*_*R*_^*2*^, cf. Figure 2). The uncertainty with weight zero or one is almost twice as high as the uncertainty with weight 0.5. This holds for any amount of accumulated evidence *n*, reflecting the number of Gaussian data points from each evidence stream agents have seen. The figure is almost scale-free, i.e., visually indistinguishable for different values of *n* unless the prior variance is very small. Note that the figure is symmetric around weight 0.5.

Our second result (*Result 2*, Supplementary Materials) is that there can be uncertainty disagreement in the pristine case as well. If agents agree that wild horses should be culled, but the researcher perceives the policymaker as having an unwarranted level of confidence in her decision, we have an example of uncertainty disagreement. The policymaker, though, may be more certain because she has more evidence available to her (such as evidence on the values held by the general public towards the presence of horses), which gives additional support to the decision (see Fig. 4). In an extreme case where the researcher discounts entirely one stream of evidence in favor of another, while the policymaker weights each stream equally, the policymaker is almost twice as certain. We provide a detailed proof of why this happens in the Supplementary Materials, but roughly, the idea is that an agent who weights the streams equally views herself as having received twice as much information as an agent who completely discounts one of the streams.

Evidence streams are not narrowly defined; they can denote anything from natural or social science findings, to local and Indigenous knowledge, or anecdotal or story-based evidence. While one agent may consider something to be ‘evidence’ that the other considers outside of ‘evidence’ or insufficiently ‘rigorous’ (formally: assigns zero weight), that is not the only difference that we are discussing here. Different weights could also result from different value judgments, e.g., about the relative importance of conserving the natural ecosystem, horse welfare, tourism values, and so on. Furthermore, different weights could result from different roles, e.g., a scientist may (legitimately) perceive her role to be to provide advice based only on scientific evidence within her discipline ^15,16^, whereas a policymaker needs to consider a broad range of factors.

**Figure 5:**
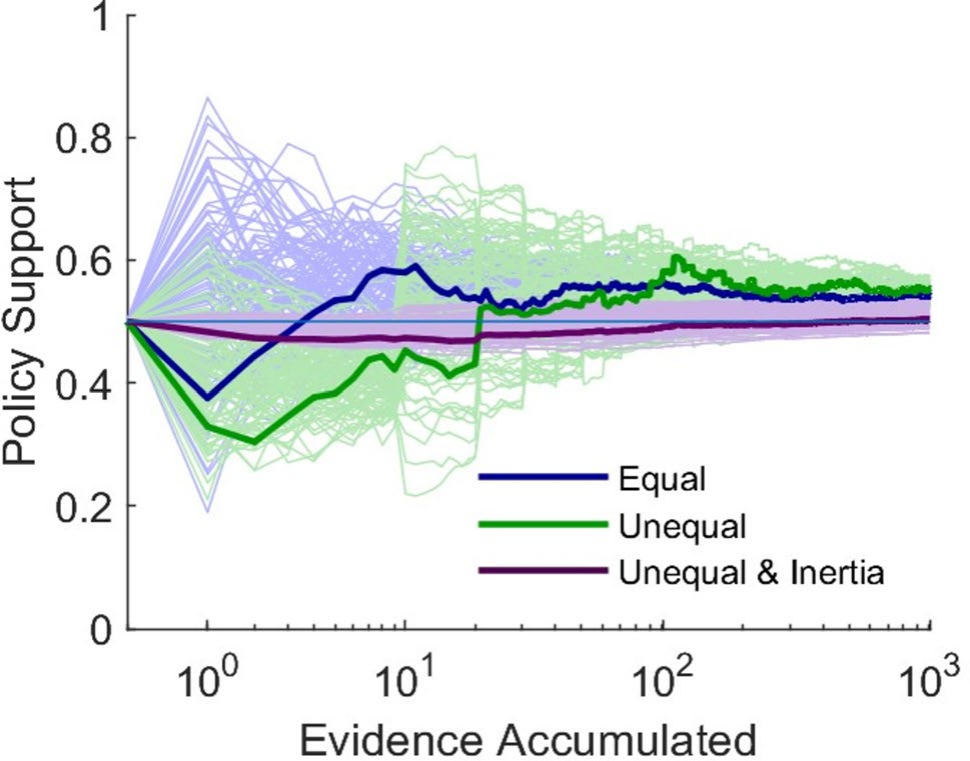
Learning at Different Speeds. This figure shows a hundred paired simulation runs in which an agent, considered under three scenarios (blue, green, purple), sees Gaussian data from two evidence streams (*μ*_*X*_ = 0.8 and *μ*_*Y*_ = 0.2 are unknown to the agent, *σ*_*X*_ = *σ*_*Y*_ = 0.25 known), which are weighted equally. On the *x*-axis, evidence accumulates as measured by the number of Gaussian data points that agents have seen from evidence stream *E*_*Y*_ (on a log scale). In the blue scenario, evidence stream *E*_*X*_ generates data at the same rate as evidence stream *E*_*Y*_. In the green and purple scenarios, evidence accumulates for *μ*_*X*_ slowly, at ten percent of the speed that evidence accumulates for *μ*_*Y*_. In just the purple scenario, there is inertia in the agent's updating her assessment of the policy.

As noted above, while it does not matter in the long run, learning in the medium run may be affected by the relative speeds with which the two evidence streams generate data. When different evidence streams come in at different rates, reflecting different methodologies’ comparative costs or paces of production and different levels of uncertainty, the faster evidence stream has more impact in early policy decisions (see Fig. 5). This is of concern where policy decisions made early set precedent for later decisions, and inertia leads us to keep doing as we have done before. This was evident early in the COVID-19 pandemic ^28,29^. For example, policies on facemasks were dictated by early research that was equivocal as to whether the virus was airborne, and the policy impact of that equivocal research lasted beyond the release of research that demonstrated more certainty ^30^.

### Faulty and faultless disagreement

In at least some of the examples above, a researcher and a policymaker weight the evidence streams differently in a way that is permissible and justifiable. Where a policy disagreement or support disagreement results purely from agents weighting streams differently (which is possible per *Result 1*) and the weights are permissibly held, we have a faultless disagreement—nothing has gone wrong with knowledge utilization in the EBPM process. Likewise, if the policymaker legitimately considers some evidence whereas the researcher legitimately ignores it, this may result in a faultless uncertainty disagreement (this is possible per *Result 2*). Nevertheless, in such cases, the researcher may think the policymaker has misunderstood the evidence she provided and characterize the outcome as a failure of adequate knowledge utilization within the EBPM process ^4^.

This is not suggesting that all disagreements in EBPM are faultless, as, first, different weightings are not always permissibly held ^31^, and second, disagreement may result from factors other than different weights that do imply fault, e.g., misunderstanding of evidence. For example, if a policymaker or decision-maker misunderstands the evidence and implements a wild horse cull policy which she would not have adopted given a reasonable understanding of the evidence, researchers would correctly assess this outcome as a failure of adequate knowledge utilization. The non-pristine case of our model highlights this by showing how systematic bias or misunderstanding in the interpretation of evidence can produce any of our three kinds of disagreement.

The pristine case and the non-pristine case highlight two different potential causes of disagreement: different weightings of the relevance of evidence (faultless in some cases) and misunderstanding of evidence. So, when researchers recommend that wild horses be managed with a cull, but a policymaker acts differently, researchers may accuse policymakers of misunderstanding or ignoring the evidence and allege a failure of adequate knowledge utilization. As we have shown, this is not necessarily the case—the disagreement may be faultless. From the perspective of policy evaluation, it is crucial to be able to tell these cases apart: whether the disagreement between researchers and policymakers is faultless or faulty determines whether adequate knowledge utilization has occurred. A sound understanding and legitimate weighting of evidence is a necessary, if not sufficient, condition for meaningfully basing policy on evidence.

Our model shows that, in the majority of cases, this debate cannot be settled. Our third result (*Result 3*, Supplementary Materials) says that in the absence of detailed information about how policymakers have interpreted and weighted the evidence (i.e., in the absence of transparency of reasoning as discussed below), faulty and faultless policy disagreements are empirically indistinguishable.

## Implications for policymakers

EBPM is widely recognized as aimed at providing greater transparency, accountability and consistency in decision-making ^32,33^, with transparency being a necessary condition of accountability ^34^. Our results indicate that, to make good on this condition, EBPM requires a distinctive form of transparency, which we call *transparency of reasoning*, to determine what type of disagreement exists and whether a disagreement is based on fault*.* Policymakers need to be transparent about not only what they consider the most decisive evidence, but about the totality of considered evidence together with some measure of how strongly that evidence supports the policy. While transparency is not always unconditionally desirable ^35^, and while our proposal will not solve all issues in EBPM, transparency of reasoning will improve understanding of disagreements and enable the evaluation of success or failure of knowledge utilization in EBPM. Without transparency of reasoning, the process of EBPM cannot be scrutinized to ensure policy was supported by evidence and adheres to other principles of good governance, or whether a decision was erroneous or biased. Further, we hypothesize that making such scrutiny possible is an important precondition of building and repairing trust in EBPM ^24^.

Transparency of reasoning is very rarely integrated into laws and decision-making policies, particularly for operational policies at the local level. Instead, transparency is usually implemented through legal incorporation into Freedom of Information (FOI) policies ^36^, formalized stakeholder consultations and feedback, and informal communications such as media releases, speeches and conferences. Decisions are typically made without extended (or any) reasons, and interested parties such as researchers may have to delve into multitudes of documents obtained under the FOI process to gain a sense of the reasons behind a decision. For example, when a decision was made to implement shark drumlines in Western Australia, the decision-maker was only required to state limited reasons for that decision. The decision-maker noted ‘substantial public concern’ about water safety and anecdotes that tourism income was suffering ^37^. If a researcher ultimately disagreed with the decision to proceed with a policy of drumlines, but the decision-maker had evidence to support public sentiment and a decline in tourist income, there could be faultless disagreement. In this case, however, once FOI requests were conducted and analyzed, researchers could legitimately shift the disagreement to one of fault, as the evidence to support public sentiment turned out not to exist ^38^. This process is time-consuming, costly, and arguably not in the spirit of transparency and accountability associated with EBPM, potentially resulting in distrust between agents.

Instead, and ideally, transparency of reasoning would be akin to the detailed reasoning provided by common law judges. Judicial reasoning is known to be, both practically and in legal philosophy, a practice that furthers transparency, accountability and participation, all goals that are shared with EBPM. Judicial reasoning expresses legal reasoning in a way that describes the actual reasoning used to make the decision ^39^. However, full judicial-type reasoning is impractical in policymaking for reasons of efficiency ^40^, ethics ^39^, and because the institutional setting of judicial reason-giving is, in key ways, fundamentally different to that of policymaking ^41^.

A more pragmatic approach to implementing transparency of reasoning would involve decision-makers indicating the evidence considered and the overall strength of the evidence in the decision-making process on a graded scale. This is akin to calls for ‘evaluation’ of evidence ^42^, although we are more proscriptive in our recommendation. For example, this process goes beyond listing evidence presented, considered or accepted, as is sometimes required ^34,43^, to include that the decision maker categorize the level of support (strong, moderate or weak) that evidence lends to a particular policy. Further research is required on how best to implement transparency of reasoning in a policy setting.

Without this type of transparency, a stakeholder cannot know whether there is a (faulty) misinterpretation of evidence, a (faulty or faultless) difference in evidence weighting, or something more sinister that we have not modeled here. However, even where disagreement is faultless, important questions can be asked about the procedures surrounding EBPM. For example, if a researcher disagrees with a policymaker that anecdotal evidence of the values or preferences of a silent majority should be given substantial weight, policy stakeholders may reconsider what is included as policy-relevant evidence.

Transparency of reasoning should also encourage greater co-production of policy between researchers and policymakers as questions and answers can be developed throughout the process rather than critique provided at the end of the process ^44,45^. Besides helping to open up the EBPM process, making it less opaque and more accessible, there will be more materials available to stimulate conversation among both sides. Scientists might even be asked to help write descriptions of evidence used and weighted as relevant in policy. True EBPM should welcome this openness.

## Implications for researchers

Not all disagreements are the fault of policymakers. Some are no one’s fault. Knowing the ways faultless disagreement emerges can lead researchers to more productively engage in EBPM. Both policymakers and researchers have roles in the EBPM process, with increasing expectations that researchers engage more fully in translating research for impact ^46,47^. Ensuring research is transparent is often discussed in the EBPM literature ^33,48,49^, but researchers engaged in EBPM should also make their best effort to ensure that the evidence is interpreted reasonably and the uncertainty is appropriately represented.

Concerning uncertainty, researchers should be aware that there is a real risk of miscommunication. For instance, a researcher may maintain a finding that wild horses are ecologically destructive, but can simultaneously express uncertainty as to whether that finding dictates a cull. Instead, she may express that the finding could lead to any number of management options. Another example is where the evidence includes assumptions that limit its applicability, and policymakers interpret those research limitations as uncertainty regarding a policy decision. Good communication is required so that lack of confidence is not construed by policymakers as a veiled attempt to secure more funding ^50^.

Co-production can help to address a disconnect between research and policymakers’ needs or demands, which can arise from multiple angles: from policymakers’ lack of skill, interest, or incentive to engage researchers in what they require from evidence, or researchers’ lack of knowledge about policymakers’ priorities at local, national, regional, or global levels, and lack of a ‘big picture’ outlook regarding the fit of the research into policymakers’ and society’s needs. Further research is warranted on the extent to which co-production of policy might bring weights into closer alignment or increase transparency.

The possibility of faultless disagreement means that not all debates should aim at consensus, whether between researchers and policymakers or among researchers. A co-production process which aims not at agreement, but at deliberation and elaboration of the policy alternatives and their various impacts, may be more suitable to EBPM ^51^. Philosophers have outlined conditions for having productive scientific debates in these contexts, where empirical evidence is not by itself decisive: e.g., the debate should take place in publicly recognized venues, criticism must be taken seriously, the standards of evaluation must be public, and a presumption of intellectual equality applies to the participants ^52,53^.

Finally, our findings regarding different learning speeds (see Fig. 5) suggest recommendations for researchers whose methodology takes longer to implement. Researchers whose methodology is slower may want to contextualize their results and explain how their new data should have been taken into account in previous policymaking, or be aware that they may have to push more strongly to get policymakers to take that data into account. This could also bolster advocacy for slower or costlier methodologies in EBPM, e.g., randomized controlled trials, where such methods are independently thought feasible.

## Conclusion

By greatly simplifying real-world matters, our model highlights how faultless disagreement can arise and how it can be indistinguishable from faulty disagreement, with particular application to EBPM. We have only considered two agents and two streams of evidence, we have not considered external actors influencing the policy process, interactions between local and national government bodies, weighting multiple policies against each other, how evidence streams are selected, and other factors, though we would expect similar conclusions from a more realistic model. The chosen simplifications cut through the complexity inherent in social life to show how factors that underlie any attempt at EBPM—evidence, the weights agents put on it, and potential misinterpretation—can produce both faultless and faulty disagreement.

Our findings are applicable to a wide range of scenarios in which disagreements involving knowledge utilization may arise, but we have shown that they pose a particular roadblock for the EBPM movement and controversies over policymaking. How do we resolve evidence-based disagreement over wild horse culling, shark drumlines, or facemask policies? Transparency of reasoning will not resolve these disagreements, but is a necessary first step. To know whether a policy is evidence-based, we need to know whether the evidence has been understood and properly incorporated. With recent increases in EBPM, implementing measures to ensure transparency of reasoning and open, productive debates are of utmost importance.

## Supplementary Information

Supplementary Information.

## Data Availability

All data and computer code used to generate figures and any other results in this paper is available in the Supplementary Materials.
