# Supplementary material for: A model of faulty and faultless disagreement for post-hoc assessments of knowledge utilization in evidence-based policymaking
Source: Sci Rep. 2024 Aug 9;14:18495. doi: 10.1038/s41598-024-69012-3 (PMC11316112; doi:10.1038/s41598-024-69012-3)
Supplement: Supplementary file 1 — Supplementary Information. [file 41598_2024_69012_MOESM1_ESM.pdf]

Supplementary Materials for

A Model of Faulty and Faultless Disagreement for Post-Hoc Assessments of Knowledge Utilization in Evidence-Based Policymaking

Remco Heesen Hannah Rubin Mike D. Schneider
Katie Woolaston Alejandro Bortolus
Emelda E. Chukwu Ricardo Kaufer Veli Mitova
Anne Schwenkenbecher Evangelina Schwindt
Helena Slanickova Temitope O. Sogbanmu
Chad L. Hewitt

Supplementary Methods: The Model and the Results

Disagreement about the policy without bias

Our modeling framework starts from the assumption that there is some policy proposal at hand. With regard to this policy proposal, a binary decision needs to be made (e.g., adopt the policy or do nothing). This decision is intended to be made on the basis of evidence. We introduce the (continuous) variable P to represent the amount of evidential support for either side of this decision. In doing so, $P = 1$ is intended to be interpreted as overwhelming support for an affirmative decision and $P = 0$ as overwhelming support for a negative decision (one could loosely interpret P as the probability that the affirmative decision is better than the negative decision, though with the caveat that the model can in principle produce states where an agent believes P to exceed one or be negative).

The evidential support for the decision at hand is a weighted average of two components, with each component representing a type of relevant information or evidence or signal that bears on the decision. The two components are μ_X and μ_Y (where we assume $\mu_X, \mu_Y \in [0,1]$), which represent the information that can be learned from two evidence streams, E_X and E_Y respectively. As noted in the main text, the two types of evidence can represent a range of different things: they may distinguish traditionally recognized scientific evidence from marginalized ways of knowing; or natural science evidence from social science evidence (where the latter is understood to bear primarily on questions concerning policy implementation); or all 'hard' evidence (in natural and social sciences) from economic modeling or forecasting; or so on. For the sake of concreteness, below we sometimes refer to μ_X as the (objective) scientific support for the policy and μ_Y as the amount of (objective) non-scientific support for the policy.

We assume also the existence of two agents: the researcher R and the decision maker D. Note that the names for the agents are just labels — the model can equally be interpreted as being about two researchers, two decision makers, a decision maker and a knowledge broker, a researcher and a member of the public, etc. The important point is that the two agents can, and typically will, weight the importance of the two components of evidential support differently. In fact, in the pristine case this is the only difference between them. We represent this as follows:

$$P_R = w_R \mu_X + (1 - w_R)\mu_Y, \tag{1}$$
$$P_D = w_D \mu_X + (1 - w_D)\mu_Y. \tag{2}$$

That is, from R's perspective, the evidential support for the policy is P_R, a linear average of μ_X and μ_Y weighted by $w_R \in [0,1]$, whereas for D, evidential support is P_D controlled by a weight parameter $w_D \in [0,1]$ (see Fig. 1). As an extreme example, R might be unaware of or uninterested in the non-scientific support for the policy ($w_R = 1$), whereas D needs to take both sides very seriously ($w_D = 1/2$).

The two agents face uncertainty about the value of the two parameters μ_X and μ_Y, but they receive probabilistic signals about their value. We distinguish between a highly general version of the model and a more specific instantiation. For the general case, assume two distribution functions F_{μ_X} and F_{μ_Y}. At each time step, a signal $E_X \mid \mu_X \sim F_{\mu_X}$ and another signal $E_Y \mid \mu_Y \sim F_{\mu_Y}$ are released to the agents (that is, conditional on the true value of

μ_X, the signal E_X follows the distribution specified by F_{μ_X}, and likewise for E_Y). So, after n time steps, the agents have access to $2n$ data points that bear on the parameters. We assume each data point to be probabilistically independent of the other $2n-1$ data points, conditional on the true values of the parameters. We also assume that the model is identifiable for μ_X and μ_Y in the sense specified in the Bernstein-von Mises theorem (theorem 10.1 in [54]), that is, it is differentiable in quadratic mean and a relevant sequence of uniformly consistent tests exists (note that these assumptions are satisfied for the specific case analyzed below).

For the specific case, we add distributional assumptions (i.e., we give specific distributions for F_{μ_X} and F_{μ_Y}) as follows: $E_X \mid \mu_X \sim N(\mu_X, \sigma^2)$ and $E_Y \mid \mu_Y \sim N(\mu_Y, \sigma^2)$. That is, the signals are normally distributed around the parameter of interest, with (shared) variance $\sigma^2 > 0$ which is assumed to be known.

We assume the two agents operate according to standard Bayesian decision theory and Bayesian statistics. This means they start with prior beliefs about the two parameters and the resulting evidential support for the policy. These take the form of probability distributions for each parameter indicating how likely they think each potential value is (we assume these two distributions are probabilistically independent), and similarly a probability distribution for the evidential support for the policy constrained by the linear equations given above. We assume identical priors for the policy: $\pi_R(P_R) \sim \pi_D(P_D)$. Due to the potentially different weightings w_R and w_D, in general this means that the priors for μ_X and μ_Y are not identical across agents.

For the general case, assume that these priors are absolutely continuous in a neighborhood of the true values of μ_X and μ_Y (and therefore also P_R and P_D). For the specific case, assume normally distributed priors with identical prior means $m \in [0,1]$ and variance chosen such that the assumption $\pi_R(P_R) \sim \pi_D(P_D)$ is satisfied:

$$\pi_R(\mu_X) \sim N(m, s^2/w_R) \quad \text{and} \quad \pi_R(\mu_Y) \sim N(m, s^2/(1-w_R)).$$

Since P_R is a convex combination of μ_X and μ_Y per equation (1), it follows that P_R also follows a normal distribution with mean m and variance s^2. Likewise for D:

$$\pi_D(\mu_X) \sim N(m, s^2/w_D) \quad \text{and} \quad \pi_D(\mu_Y) \sim N(m, s^2/(1-w_D))$$

entails that $\pi_D(P_D) \sim N(m, s^2) \sim \pi_R(P_R)$.

For the specific case, we can specify the posteriors explicitly. Let E_n be the total data seen after n time steps, i.e., E_n is a vector containing n draws from E_X and n draws from E_Y, all probabilistically independent. Let $\bar{x}$ be the average of the n draws from E_X, and $\bar{y}$ the average of the E_Ys. Then the posteriors are:

$$\begin{aligned}
\pi_R(\mu_X \mid E_n) &\sim N\left(\frac{ns^2\bar{x} + w_R\sigma^2 m}{ns^2 + w_R\sigma^2}, \frac{s^2\sigma^2}{ns^2 + w_R\sigma^2}\right)\\
\pi_R(\mu_Y \mid E_n) &\sim N\left(\frac{ns^2\bar{y} + (1-w_R)\sigma^2 m}{ns^2 + (1-w_R)\sigma^2}, \frac{s^2\sigma^2}{ns^2 + (1-w_R)\sigma^2}\right)\\
\pi_D(\mu_X \mid E_n) &\sim N\left(\frac{ns^2\bar{x} + w_D\sigma^2 m}{ns^2 + w_D\sigma^2}, \frac{s^2\sigma^2}{ns^2 + w_D\sigma^2}\right)\\
\pi_D(\mu_Y \mid E_n) &\sim N\left(\frac{ns^2\bar{y} + (1-w_D)\sigma^2 m}{ns^2 + (1-w_D)\sigma^2}, \frac{s^2\sigma^2}{ns^2 + (1-w_D)\sigma^2}\right)
\end{aligned}$$

The posteriors for the policy can be specified explicitly as well, though the expressions are a bit unwieldy. The posteriors $\pi_R(P_R \mid E_n)$ and $\pi_D(P_D \mid E_n)$ are both normal distributions (because linear combinations of independent normal distributions are normal) with mean and variance given by the usual formulas for linear combinations of independent random variables:

$$\begin{aligned}
\mathbb{E}_{\pi_R}[P_R \mid E_n] &= w_R\mathbb{E}_{\pi_R}[\mu_X \mid E_n] + (1-w_R)\mathbb{E}_{\pi_R}[\mu_Y \mid E_n],\\
\operatorname{Var}_{\pi_R}[P_R \mid E_n] &= w_R^2 \operatorname{Var}_{\pi_R}[\mu_X \mid E_n] + (1-w_R)^2 \operatorname{Var}_{\pi_R}[\mu_Y \mid E_n],
\end{aligned}$$

and similarly for D. We define μ_R, μ_D, σ_R^2, and σ_D^2 as short-hands for the posterior means and variances:

$$\begin{aligned}
\mu_R &= \mathbb{E}_{\pi_R}[P_R \mid E_n], & \sigma_R^2 &= \operatorname{Var}_{\pi_R}[P_R \mid E_n],\\
\mu_D &= \mathbb{E}_{\pi_D}[P_D \mid E_n], & \sigma_D^2 &= \operatorname{Var}_{\pi_D}[P_D \mid E_n].
\end{aligned}$$

Note that, technically, these short-hands should be indexed to n, the amount of evidence seen by the agents, but we drop this index for notational convenience.

For establishing the first result, the key observation is that as n gets large, the posterior means for μ_X and μ_Y approach the average of the data ($\bar{x}$ and $\bar{y}$ respectively), and the variance goes to zero, i.e., $\pi_R(\mu_X \mid E_n)$ and

$\pi_D(\mu_X \mid E_n)$ converge in probability to $\bar{x}$, and $\pi_R(\mu_Y \mid E_n)$ and $\pi_D(\mu_Y \mid E_n)$ converge in probability to $\bar{y}$. Moreover, $\bar{x} \mid \mu_X \sim N(\mu_X, \sigma^2/n)$ and $\bar{y} \mid \mu_Y \sim N(\mu_Y, \sigma^2/n)$, which means that the sample averages converge in probability to the true value of the parameters. So, in the long run learning is successful in the sense that the posteriors will converge to the true values of the parameters. In symbols:

$$\pi_R(\mu_X \mid E_n) \xrightarrow{p} \mu_X \xleftarrow{p} \pi_D(\mu_X \mid E_n),$$
$$\pi_R(\mu_Y \mid E_n) \xrightarrow{p} \mu_Y \xleftarrow{p} \pi_D(\mu_Y \mid E_n).$$

For the posteriors for the policy, it follows that they converge as well:

$$\pi_R(P_R \mid E_n) \xrightarrow{p} w_R\mu_X + (1 - w_R)\mu_Y = P_R,$$
$$\pi_D(P_D \mid E_n) \xrightarrow{p} w_D\mu_X + (1 - w_D)\mu_Y = P_D.$$

So, in the pristine case learning is successful in the sense that with sufficient data the agents will come to hold beliefs about P that match the true value of what they set out to estimate. Note that the claim here is that the entire posterior distributions converge in probability to point masses; this entails the weaker claim that the posterior means also converge (μ_R to P_R and μ_D to P_D).

In particular, if $P_R < 1/2$ and $P_D > 1/2$ (or vice versa), which can happen if μ_X and μ_Y are on different sides of 1/2 and the weights w_R and w_D are different from each other, then the probability of policy disagreement between the agents approaches one as n gets large. Since we started with identical priors, the probability of policy disagreement (or any other kind of disagreement discussed in the main text) before seeing any data is zero. So, we see that the probability of policy disagreement increases with data. In fact, with a bit of extra work it can be shown that the probability of policy disagreement increases monotonically, i.e., for any n, the probability of disagreement given E_{n+1} is higher than the probability of disagreement given E_n. (Here, we assume that agents base their recommendation or decision on the posterior mean, though since they start from identical prior distributions and converge to a point-mass distribution the same conclusion will hold for any other reasonable metric that the agents might use, such as basing their decision on the median or more generally a probabilistic threshold. We also assume that there is no prior reason to favor a positive or negative decision, so the decision threshold is at 1/2; this immediately generalizes to the thresholds identified in the next section.) All of this is illustrated in Fig. 3.

A similar argument goes through in the general case. While we cannot specify the posterior distribution explicitly, due to the Bernstein-von Mises theorem (theorem 10.1 in [54]) we know that the posteriors for μ_X and μ_Y converge probabilistically on the true values of the parameters, just like in the specific case. Hence, again, $\pi_R(P_R \mid E_n)$ converges in probability to P_R and $\pi_D(P_D \mid E_n)$ converges in probability to P_D. So, again, if $P_R < 1/2$ and $P_D > 1/2$ (or vice versa), and if we assume that the two agents base their decision or recommendation on the posterior mean, then as n gets large the probability of policy disagreement between R and D approaches one, whereas at $n = 0$ the probability of disagreement is zero. This establishes the first result.

Result 1. In the pristine case, if $P_R < 1/2$ and $P_D > 1/2$ or vice versa, the probability of policy disagreement increases monotonically in n, and approaches one as n gets large. Assuming the distributions F_{μ_X} and F_{μ_Y} are continuous, support disagreement occurs in the pristine case with probability one whenever $w_R \neq w_D$ and $n \geq 1$.

The clause about policy disagreement was established in the preceding paragraph. The clause about support disagreement follows straightforwardly from the definition: we have support disagreement whenever $\mu_R \neq \mu_D$. If $w_R = w_D$ then the agents' beliefs about the evidence and the policy are always exactly the same and support disagreement never occurs. But if $w_R \neq w_D$ and $n \geq 1$, the only way to get $\mu_R = \mu_D$ is if $\bar{x}$ and $\bar{y}$ take on precisely the right values (for any specified value of one there is precisely one value of the other that yields $\mu_R = \mu_D$). Assuming continuous distributions for the data (including the specific case of normally distributed data), this is a probability zero event. Hence (some) support disagreement occurs with probability one, though the degree of support disagreement (measured as the distance between μ_R and μ_D in Fig. 3c) may vary.

Disagreement about risk to act

To do a risk analysis (in the sense used in statistics), we need to specify a loss function. Note that the terminology of 'loss function' is standard in statistics — economists and philosophers will recognize this as a utility function multiplied by negative one. Risk is then defined as expected loss, so a risk-minimizing decision is equivalent to an expected utility-maximizing decision. We have a slightly unusual case here because the variable P is

continuous (evidential support for the policy proposal can range continuously from strongly favoring to strongly disfavoring it) but the decision problem is binary (either the policy is adopted or not).

Let d_P and $d_{\neg P}$ stand for the decisions to adopt the policy or not, respectively. We start with a simple loss function L_1 which assumes that loss is linear in P, with a negative slope under d_P (if the true value of P is high and the policy is adopted, loss is low) and a positive slope under $d_{\neg P}$ (if the true value of P is low and the policy is not adopted, loss is low). This is shown in Table S1.

	$L_1(d, P)$	$L_2(d, P)$
d_P	$\ell_\alpha(1 - P)$	$\ell_\alpha(1 - P)^2$
$d_{\neg P}$	$\ell_\beta P$	$\ell_\beta P^2$

Table S1: This table shows two potential loss functions for the purpose of a risk analysis of the agents' decision-making under uncertainty. The linear loss function L_1 appears on the left and the quadratic loss function L_2 appears on the right. We assume that $P \in [0, 1]$ and $\ell_\alpha, \ell_\beta > 0$. In each case, the loss increases as the underlying evidential support for the policy is further removed from the decision taken. Thus, if the policy is implemented (d_P), smaller values of P imply larger loss, whereas if the policy is not implemented ($d_{\neg P}$), larger values of P imply larger loss.

The two slopes are given as parameters, $\ell_\alpha, \ell_\beta > 0$. Only the ratio between them matters. If $\ell_\alpha > \ell_\beta$, this means that the negative consequences of adopting the policy when this is a bad idea (P is low) are relatively large compared to the negative consequences of failing to adopt the policy when P is high. These parameters should thus be set based on the real-life stakes of the decision, especially when a 'wrong' decision is made.

To analyze the decision process from a risk perspective, we work out the empirical risk, which is the expected loss given the data for each decision.

$$\begin{aligned}
\hat{r}_R(d_{\neg P}) &= \mathbb{E}_{\pi_R}[L_1(d_{\neg P}, P_R) \mid E_n] = \ell_\beta \mathbb{E}_{\pi_R}[P_R \mid E_n], \\
\hat{r}_R(d_P) &= \mathbb{E}_{\pi_R}[L_1(d_P, P_R) \mid E_n] = \mathbb{E}_{\pi_R}[\ell_\alpha(1 - P_R) \mid E_n] \\
&= \ell_\alpha(1 - \mathbb{E}_{\pi_R}[P_R \mid E_n]).
\end{aligned}$$

We see that

$$\hat{r}_R(d_{\neg P}) \geq \hat{r}_R(d_P) \quad \text{if and only if} \quad \mathbb{E}_{\pi_R}[P_R \mid E_n] \geq \frac{\ell_\alpha}{\ell_\alpha + \ell_\beta},$$

which is just what you would expect: after seeing the data, R estimates P_R using the posterior mean, and favors adopting the policy (d_P) if the posterior mean is high, and not adopting the policy if the posterior mean is low. If the loss function is symmetric ($\ell_\alpha = \ell_\beta$) then the policy should be adopted if and only if the posterior mean exceeds one-half (we implicitly assumed this for simplicity in the previous subsection, though it makes no difference to the analysis there). If ℓ_α is larger, then it is better not to adopt the policy in case of doubt, so the threshold is higher than one-half, and vice versa if ℓ_β is larger.

The analysis is identical for D: the optimal decision is to adopt the policy if and only if the posterior mean exceeds the threshold $\ell_\alpha/(\ell_\alpha + \ell_\beta)$.

So far, everything applies to the general case, as we have not used any distributional assumptions on the priors or the data. To compare the risk for the two agents R and D, we move back to the specific case where the priors and the data are assumed to follow a normal distribution.

Suppose $\mu_X \approx 0$ and $\mu_Y \approx 0$ and n is fairly large. In that case the distribution of the average of the data ($\bar{x} \mid \mu_X$ and $\bar{y} \mid \mu_Y$) has a very small variance σ^2/n, i.e., with very high probability $\bar{x} \approx 0$ and $\bar{y} \approx 0$. This means, with very high probability,

$$\begin{aligned}
\mathbb{E}_{\pi_R}[\mu_X \mid E_n] &= \frac{ns^2\bar{x} + w_R\sigma^2 m}{ns^2 + w_R\sigma^2} \approx \frac{w_R\sigma^2 m}{ns^2 + w_R\sigma^2}, \\
\mathbb{E}_{\pi_R}[\mu_Y \mid E_n] &\approx \frac{(1-w_R)\sigma^2 m}{ns^2 + (1-w_R)\sigma^2}, \\
\mathbb{E}_{\pi_R}[P_R \mid E_n] &\approx w_R\frac{w_R\sigma^2 m}{ns^2 + w_R\sigma^2} + (1-w_R)\frac{(1-w_R)\sigma^2 m}{ns^2 + (1-w_R)\sigma^2}.
\end{aligned}$$

And similarly,

$$\mathbb{E}_{\pi_D}[P_D \mid E_n] \approx w_D\frac{w_D\sigma^2 m}{ns^2 + w_D\sigma^2} + (1-w_D)\frac{(1-w_D)\sigma^2 m}{ns^2 + (1-w_D)\sigma^2}.$$

Since n is assumed to be large, these posterior means are close to zero, or at least less than the threshold $\ell_\alpha/(\ell_\alpha + \ell_\beta)$, so both agents will choose decision

$d_{\neg P}$. As noted above, the empirical risk associated with this decision is just ℓ_β times the posterior mean, which we have just approximated with the expressions immediately above. Moreover, if we look at the denominators of the fractions, we see that, since n is large, the term ns^2 dominates these expressions, so

$$\begin{aligned}
\hat{r}_R(d_{\neg P}) &\approx \ell_\beta w_R \frac{w_R \sigma^2 m}{ns^2} + \ell_\beta (1 - w_R) \frac{(1 - w_R)\sigma^2 m}{ns^2} \\
&= \frac{\ell_\beta \sigma^2 m}{ns^2} \left(w_R^2 + (1 - w_R)^2 \right), \\
\hat{r}_D(d_{\neg P}) &\approx \frac{\ell_\beta \sigma^2 m}{ns^2} \left(w_D^2 + (1 - w_D)^2 \right).
\end{aligned}$$

We see that the empirical risk is lower for whichever agent's weights are closer to one-half. In the extreme case where $w_R = 1$ and $w_D = 1/2$ (which we also used as an example above), the empirical risk for D is only half of the empirical risk for R (the same would hold if $w_R = 0$).

So, here we have a case where the two agents agree about what should be done, but their perception of the risk involved in making that decision is different. In particular, if D considers the two evidence streams approximately equally important, whereas R heavily favors E_X, then R might perceive D to be making her decision with an unwarranted level of confidence (despite agreeing with the substance of the decision).

The same analysis goes through if $\mu_X \approx 1$ and $\mu_Y \approx 1$ and n is fairly large. Then $\bar{x} \approx 1$ and $\bar{y} \approx 1$ with high probability, so

$$\begin{aligned}
\mathbb{E}_{\pi_R}[\mu_X \mid E_n] &= \frac{ns^2 \bar{x} + w_R \sigma^2 m}{ns^2 + w_R \sigma^2} \approx \frac{ns^2 + w_R \sigma^2 m}{ns^2 + w_R \sigma^2}, \\
\mathbb{E}_{\pi_R}[\mu_Y \mid E_n] &\approx \frac{ns^2 + (1 - w_R)\sigma^2 m}{ns^2 + (1 - w_R)\sigma^2}.
\end{aligned}$$

Since both of the above expressions are close to one, the posterior mean for P_R will be close to one (since it is a weighted average of the above) and hence decision d_P will be chosen. Likewise D's posterior mean for P_D will be close to one and so D makes the same decision. The empirical risk for this decision

is

$$\begin{aligned}
\hat{r}_R(d_P) &= \ell_\alpha w_R \mathbb{E}_{\pi_R}[1-\mu_X \mid E_n] + \ell_\alpha (1-w_R)\mathbb{E}_{\pi_R}[1-\mu_Y \mid E_n] \\
&\approx \ell_\alpha w_R \frac{w_R \sigma^2 (1-m)}{ns^2 + w_R\sigma^2} + \ell_\alpha (1-w_R) \frac{(1-w_R)\sigma^2(1-m)}{ns^2+(1-w_R)\sigma^2} \\
&\approx \frac{\ell_\alpha \sigma^2 (1-m)}{ns^2}\left(w_R^2 + (1-w_R)^2\right), \\
\hat{r}_D(d_P) &\approx \frac{\ell_\alpha \sigma^2 (1-m)}{ns^2}\left(w_D^2 + (1-w_D)^2\right).
\end{aligned}$$

Again, the empirical risk is lower for whichever agent's weights are closer to one-half.

Though we only provide a full analysis for the cases where $\mu_X \approx 0 \approx \mu_Y$ or $\mu_X \approx 1 \approx \mu_Y$, we have good reason to think that, quite generally, the agent whose weights are closer to one-half will perceive the risk to be lower, at least for (moderately) large n. One way to see this is by looking at the posterior variance, another measure of the degree of uncertainty the agents perceive. For large n,

$$\mathrm{Var}_{\pi_R}[\mu_X \mid E_n] = \frac{s^2\sigma^2}{ns^2 + w_R\sigma^2} \approx \frac{\sigma^2}{n},$$

and similarly for μ_Y and for D. Hence

$$\begin{aligned}
\sigma_R^2 = \mathrm{Var}_{\pi_R}[P_R \mid E_n] &\approx \frac{\sigma^2}{n}\left(w_R^2 + (1-w_R)^2\right), \\
\sigma_D^2 = \mathrm{Var}_{\pi_D}[P_D \mid E_n] &\approx \frac{\sigma^2}{n}\left(w_D^2 + (1-w_D)^2\right).
\end{aligned}$$

So, again, the agent whose weights are closer to one-half perceives less uncertainty, up to a factor of two in the most extreme case when one agent's weights are exactly one-half and the other's are one and zero (see Fig. 4, which was generated using the exact formula for σ_R^2 with $\sigma = 2$, $s = 5$, and n ranging from 10 to 100). This is our second result.

Result 2. In the specific case (with normally distributed data and priors) of the pristine case of the model, if $w_R \neq w_D$, agents will generally differ in how uncertain they perceive their recommendation to be, as measured in the posterior variance. At its most extreme, when $w_R \in \{0, 1\}$ and $w_D = 1/2$ (or vice versa) and n is large, perceived uncertainty may be twice as large

for one agent compared to the other.

One might worry that some of the above analysis depends crucially on the assumption of linear loss. To (partially) address this worry, we briefly consider the quadratic loss function L_2, shown in Table S1.

As before, we work out the empirical risk (expected loss given the data), using the fact that for any random variable X with finite variance the second moment is a simple function of its mean and variance: $\mathbb{E}[X^2] = \mathbb{E}[X]^2 + \mathrm{Var}[X]$.

$$\begin{aligned}
\hat{r}_R(d_{\neg P}) &= \mathbb{E}_{\pi_R}[L_2(d_{\neg P}, P_R) \mid E_n] = \ell_\beta \mathbb{E}_{\pi_R}[P_R^2 \mid E_n] \\
&= \ell_\beta \mathbb{E}_{\pi_R}[P_R \mid E_n]^2 + \ell_\beta \mathrm{Var}_{\pi_R}[P_R \mid E_n], \\
\hat{r}_R(d_P) &= \ell_\alpha \mathbb{E}_{\pi_R}[(1 - P_R)^2 \mid E_n] \\
&= \ell_\alpha - 2\ell_\alpha \mathbb{E}_{\pi_R}[P_R \mid E_n] + \ell_\alpha \mathbb{E}_{\pi_R}[P_R \mid E_n]^2 + \ell_\alpha \mathrm{Var}_{\pi_R}[P_R \mid E_n].
\end{aligned}$$

We see that $\hat{r}_R(d_{\neg P}) \geq \hat{r}_R(d_P)$ if and only if

$$\frac{\ell_\beta - \ell_\alpha}{2\ell_\alpha} \mathbb{E}_{\pi_R}[P_R^2 \mid E_n] + \mathbb{E}_{\pi_R}[P_R \mid E_n] \geq \frac{1}{2}.$$

This is slightly more complicated than what we found for the linear case, but the basic idea is similar. If $\ell_\beta = \ell_\alpha$, adopting the policy (d_P) should be favored (its risk is lower) just in case the posterior mean exceeds one-half. Noting that $\mathbb{E}_{\pi_R}[P_R^2 \mid E_n]$ is necessarily positive, we see that if $\ell_\beta > \ell_\alpha$, the second moment skews the decision in favor of d_P, whereas if $\ell_\beta < \ell_\alpha$ the contribution of this term is negative, amounting to a skew in favor of $d_{\neg P}$. All this applies identically to D if all the subscript-Rs are replaced with subscript-Ds.

As before, if $\mu_X \approx 0 \approx \mu_Y$ and n is fairly large, then with high probability $\bar{x} \approx 0 \approx \bar{y}$, so the posterior mean will be low and decision $d_{\neg P}$ chosen. Previously, we estimated that in this scenario

$$\begin{aligned}
\mathbb{E}_{\pi_R}[P_R \mid E_n] &\approx \frac{\sigma^2 m}{n s^2} \left(w_R^2 + (1 - w_R)^2 \right), \\
\mathrm{Var}_{\pi_R}[P_R \mid E_n] &\approx \frac{\sigma^2}{n} \left(w_R^2 + (1 - w_R)^2 \right),
\end{aligned}$$

and similarly for D. Hence

$$\hat{r}_R(d_{\neg P}) \approx \frac{\ell_\beta \sigma^2}{n} \left(w_R^2 + (1-w_R)^2\right) \left(1 + \frac{\sigma^2 m^2}{n s^4} \left(w_R^2 + (1-w_R)^2\right)\right),$$
$$\hat{r}_D(d_{\neg P}) \approx \frac{\ell_\beta \sigma^2}{n} \left(w_D^2 + (1-w_D)^2\right) \left(1 + \frac{\sigma^2 m^2}{n s^4} \left(w_D^2 + (1-w_D)^2\right)\right).$$

Here again we observe that the agent whose weights are closer to one-half perceives lower risk. If we simplify the expressions a bit further by neglecting all terms with denominator n^2 (which are small relative to those with denominator n), we see that the variation in risk perception due to the different weights ranges up to a factor two, just as before:

$$\hat{r}_R(d_{\neg P}) \approx \frac{\ell_\beta \sigma^2}{n} \left(w_R^2 + (1-w_R)^2\right),$$
$$\hat{r}_D(d_{\neg P}) \approx \frac{\ell_\beta \sigma^2}{n} \left(w_D^2 + (1-w_D)^2\right).$$

Noise, bias, and underdetermination in the model

One potential cause of disagreement not captured in the pristine case is when the decision maker (agent D) has a different understanding of the evidential support for the policy than the researcher (agent R). Here we focus in particular on different understandings of the scientific support, that is, the parameter μ_X and the relevant data E_X.

We can think of a lot of reasons why the two agents might perceive this differently. It could be that D misinterprets or misunderstands the data (this is suggested on p. 4 of [55]). It could be that due to a lack of time or limitations of resources D sees only a subsample of the data. It could be that some third agent (such as an employee of D or a knowledge broker) selects or summarizes the data for D, potentially with some loss of fidelity.

In this section, we consider a subset of such scenarios which can be modeled as a combination of 'white noise' (some random disturbance to the signal that D receives) and systematic bias (a shift to the signal that D receives in a specific direction). Let ε_X be a random variable that captures the noise. We assume that this is added to any data that D sees. More precisely, whereas at any time step R perceives a random draw from E_X, instead D perceives a random draw from $E'_X = E_X + \varepsilon_X$. We assume that draws from ε_X are independent and identically distributed, and that draws from ε_X are independent from any of the data in E_n.

Recall that we distinguished between a specific case of the model (where the data and priors are all assumed to follow a normal distribution) and a general case without distributional assumptions. For the specific case, assume that the noise is also normally distributed: $\varepsilon_X \sim N(b_X, \tau_X^2)$. Here, b_X represents the systematic bias, and τ_X^2 represents the degree of white noise added. In particular, if $b_X = 0$ then ε_X is just white noise, with higher values of τ_X indicating more noise. For the general case, we make no distributional assumption, but we do assume that ε_X has a finite mean b_X and finite variance τ_X^2. By not specifying a normal distribution, we can accommodate a wide variety of distributions of potentially very unusual shape, restricted only by having finite mean and variance.

Consider first the case of merely white noise and no systematic bias ($b_X = 0$). The presence, absence, or size of τ_X^2 makes no difference to the application of the Bernstein-von Mises theorem, so long-term convergence is completely unaffected. In other words, while the speed of learning is delayed, its trajectory is unchanged. We still get disagreement if the true values of P_R and P_D lead to different recommendations (*Result 1* is unchanged). The consequences for *Result 2* depend on whether agent D is aware of the white noise. If she knows about it, she perceives more uncertainty than in the pristine case, potentially offsetting the lower uncertainty described in *Result 2*. If she does not know about the white noise, she perceives less uncertainty (per *Result 2*), but might in fact face as much uncertainty as agent R or more.

Now consider the systematic bias b_X. Here we assume that D is not aware of the existence or size of the bias (if she knew its size, she could easily correct for the bias and proceed as if it were not there). The bias itself is simply a positive or negative constant shifting her perception of the evidence stream E_X either in favor of or against the policy.

The most important consequence of the presence of systematic bias is that the mean of the scientific signal as perceived by D is $\mu_X + b_X$, which is therefore also the value that her posterior distribution converges to. This means that, with regard to the policy, D converges to

$$P'_D = w_D(\mu_X + b_X) + (1 - w_D)\mu_Y. \quad (3)$$

Compared to the pristine case, this introduces new possible outcomes. Most notably, we could have disagreement due to systematic bias where there would not have been disagreement in the pristine case (so $P_R < 1/2$, $P_D <$

$1/2$, $P'_D > 1/2$, or similarly with all three inequalities reversed) and we could have agreement due to systematic bias where there would have been disagreement in the pristine case (so $P_R < 1/2$, $P_D > 1/2$, $P'_D < 1/2$, or all three reversed). The latter would be a case where the decision maker arguably should disagree with the researcher (assuming the different weights reflect a legitimate difference in value judgments) but does not due to the effects of the systematic bias. Here, the combination of different value judgments and misinterpretation canceling each other out might lead to the misinterpretation going unnoticed.

Now suppose we have observed a case of disagreement between the researcher and the decision maker. Let us assume we know all of the evidence (μ_X and μ_Y) and we know how much weight the researcher thinks the scientific evidence holds for the policy statement (w_R). We can think of this either from the researcher's point of view — they have given the decision maker good evidence, why does the decision maker not heed it? — or from the point of view of the public — they have seen the scientific evidence provided, and heard how important it is, why does the decision maker not listen?

One interesting thing to note is that, in the framework provided and in the absence of further information about the decision maker's reasoning process, it is impossible to tell whether the decision maker is misinterpreting the evidence, i.e., $b_X \neq 0$, or simply has a different weighting scheme to the researcher, i.e. $w_D \neq w_R$, or both. How can we see this?

Consider the world in which the decision maker has no bias but weights the evidence differently from the researcher. The decision maker's evaluation of the policy is given by the following equation, identical to equation (2) above:

$$P_D = w_D \mu_X + (1 - w_D)\mu_Y \tag{4}$$

In this possible world, any disagreement we observe must be due to differing weights.

Now consider the world in which the decision maker weights the evidence the same as the researcher, but also has some bias in their interpretation of evidence. Their evaluation of the policy is given by the following equation, which is found by setting $w_D = w_R$ in equation (3) above:

$$P'_D = w_R(\mu_X + b_X) + (1 - w_R)\mu_Y \tag{5}$$

In this possible world, any disagreement must be due to the decision maker's misinterpretation of the scientific evidence.

Assuming we regard disagreements due to different weights (different value judgments) as at least potentially legitimate and disagreements due to bias or misinterpretation as problematic, we would like to know in a given case which causal factor is responsible for the decision maker's decision. Unfortunately, in the setup we have provided, where only the evidence (μ_X and μ_Y), the researcher's weights (w_R and $1 - w_R$) and the final judgments of evidential support (P_R and either P_D or P'_D) are known, it is impossible to tell. We can always find b_X and w_D to make the two possible worlds indistinguishable, i.e., $P_D = P'_D$:

$$w_D\mu_X + (1 - w_D)\mu_Y = w_R(\mu_X + b_X) + (1 - w_R)\mu_Y.$$

Solving for b_X yields:

$$b_X = \frac{1}{w_R}(w_R - w_D)(\mu_Y - \mu_X) \tag{6}$$

Or, in terms of w_D:

$$w_D = w_R\left(1 - \frac{b_X}{\mu_Y - \mu_X}\right) \tag{7}$$

Since w_R, μ_Y, and μ_X are fixed constants, this gives us, for any speculated bias b_X, a way to explain the decision maker's choice in terms of a weighting of evidence, w_D, different from the researcher. We cannot tell whether they are misinterpreting evidence or whether they simply weight the evidence differently just by the observation that there is disagreement; we need more information about the decision making process.

Result 3. Given μ_X, μ_Y, w_R, and the final judgments of evidential support (P_R and either P_D or P'_D), it is impossible to tell whether any disagreement between the two agents is due to different weights ($w_D \neq w_R$) or bias ($b_X \neq 0$); that is, we can find values for w_D and b_X consistent with either explanation.

One might object that the above assumes w_D can take any value, while we ought to impose rationality constraints on the weights, i.e., that weights are between 0 and 1. However, this does little to ameliorate the issue; the problem of distinguishing between the world in which bias explains disagreement and the world in which different weightings explains disagreement remains. To see why this is the case, consider under what conditions $0 \leq w_D \leq 1$ is met. Given the proposed rationality constraints of weightings, we know that

w_R will be between 0 and 1, so from equation (7) we can see that $0 \leq w_D \leq 1$ whenever $\frac{w_R - 1}{w_R} \leq \frac{b_X}{\mu_Y - \mu_X} \leq 1$.

First, we can show that $\frac{b_X}{\mu_Y - \mu_X}$ will always be positive in cases of disagreement. There are two ways disagreement can manifest: either the researcher approves of P but the decision maker does not or the decision maker approves of P but the researcher does not. In the first case, if it were possible to explain disagreement in terms of different weightings, and we assume as before that this means the decision maker puts more weight on the non-scientific evidence and hence less weight on the scientific evidence ($w_D < w_R$), then we must have $\mu_Y < \mu_X$ and therefore $\mu_Y - \mu_X < 0$. If we were to explain the disagreement in terms of bias, this bias would need to be negative (the decision maker interprets the evidence E_X as less in favor than the researcher does), so $b_X < 0$. Therefore, $\frac{b_X}{\mu_Y - \mu_X}$ is positive since both numerator and denominator are negative. By similar reasoning, in the second case, we have $\mu_Y - \mu_X > 0$ and $b_X > 0$ (as decision maker interprets E_X as more in favor than the researcher) and again $\frac{b_X}{\mu_Y - \mu_X}$ is positive. Since $\frac{w_R - 1}{w_R} \leq 0$, this means the inequality $\frac{w_R - 1}{w_R} \leq \frac{b_X}{\mu_Y - \mu_X}$ is met.

Second, the requirement that $\frac{b_X}{\mu_Y - \mu_X} \leq 1$ amounts to either $\mu_X + b_X \leq \mu_Y$ (if $\mu_Y > \mu_X$) or $\mu_X + b_X \geq \mu_Y$ (if $\mu_Y < \mu_X$). In either case, the requirement is effectively that the bias it would take to explain the disagreement is not so extreme that it pushes the decision maker's interpretation of the evidence stream E_X beyond what stream E_Y says. It is certainly possible for such extreme biases to exist, but there are reasons to think their impact on this underdetermination result is minimal. At worst, it means that sometimes bias is the only possible explanation of disagreement (i.e., there is no underdetermination), but this can only arise in relatively extreme cases of disagreement. Moreover, in practice we likely will have only qualitative, not precise quantitative information about the evidential support the decision maker claims to have for the policy decision. In these cases, there will not be enough information even to tell whether the disagreement is relatively extreme or not, so we are again in a situation of underdetermination regarding its cause. Finally, we note that the problem of underdetermination grows worse when we allow for the joint possibility of bias and inequivalent weights.

Supplementary Methods: The Model with Variable Learning Speeds

Finally, we briefly study computationally the effects of variable learning speeds across different evidence streams, as they affect learning in the medium run (that is, before enough evidence is seen that limiting behavior dominates the analysis). So: consider 'high pressure' streams, corresponding to evidence gathered over kinds of research that enjoy fast or inexpensive methodologies, compared to 'low pressure' streams (the compliment of the former). Suppose that high pressure streams send an agent H data points for every low pressure stream data point they receive. So, a high pressure stream captures faster learning about one kind of evidence, by an integer factor $H > 1$, compared to slow learning about another kind of evidence along a low pressure stream. In such a scenario, at all points in learning, an agent's estimation of how the high pressure stream evidence speaks to the policy decision is a higher fidelity signal of the underlying 'true' parameter, compared to her estimation of how the low pressure stream evidence speaks to the decision. We expect the consequence of this to be that, at least in the medium run, assessments of the binary policy decision will tend to favor the high pressure evidence stream, relative to the weights placed on those streams.

We consider a single agent learning for 1000 time-steps under three scenarios (each simulated 100 times, see Data S1 for code). This agent weights the two evidence streams equally and begins with prior ambivalence about the policy decision ($m = 0.5$, $s^2 = 0.1$). The true parameters she is learning are $\mu_X = 0.8$ and $\mu_Y = 0.3$ respectively, with standard deviation $\sigma = 0.25$ (hence, in the long run, the posterior converges towards 0.55). The first scenario (shown in blue in Fig. 5) corresponds to an agent exactly like those analysed in prior sections: equal rates of learning along both evidence streams. In the second scenario (green in Fig. 5), E_X comes along a low-pressure stream (slow learning) and E_Y comes along a high-pressure stream (fast learning), with $H = 10$. Here, the agent only gets one draw from E_X for every tenth draw from E_Y. Note that simulation runs are paired, so for each run in the first scenario there is a run in the second scenario where the agent sees exactly the same data from evidence stream E_Y and a subset of the data from evidence stream E_X.

Comparing 'equal' to 'unequal' learning speeds shows us what happens were an agent to get the same evidence, but receive evidence from one of

the streams at a slower pace. Note in Fig. 5 how, early in learning, there is a tendency for the agent to draw a more negative policy assessment (green lines are typically below blue lines), reflecting that her estimate of μ_Y is a higher fidelity signal of the true parameter value 0.3, which gets washed out later in learning.

If — as strikes us as plausible (see, e.g., [28,29]) — there is inertia in agents' combining and weighting their estimates of the counsels of each of the different bodies of evidence in order to arrive at their assessment of the policy decision, we get a memory effect: whatever is ultimately counseled in the limit by the body of high pressure stream evidence stays over-represented in the expected outcomes of early assessments of the policy decision based on noisy estimates for long times (i.e., as noise in the learning process diminishes). In Fig. 5, this is visualized in purple, where inertia is modeled as a convex combination in each round of updating of: an average of all prior policy assessments and the current assessment. (We have used an extreme value of 0.9 weighting prior assessments, for a weighting of 0.1 to the current assessment, to draw out visually the memory effect.) Note that, in the infinite limit, even the purple will agree with blue; again, the effects discussed here are exclusively relevant in the medium run of learning.

The observations in this section motivate future investigations about the impacts of various features of science and policymaking during EPBM, such as different existing incentives to focus on some relevant methodologies to the marginalization of others. Of particular interest is the situation where the ratio of pressures is not imagined as fixed at a factor H.

Supplementary References

54. van der Vaart, A. W. *Asymptotic Statistics.* (Cambridge University Press, 1998). doi:10.1017/CBO9780511802256.

55. Vickery, J. *et al.* Challenges to evidence-informed decision-making in the context of pandemics: qualitative study of COVID-19 policy advisor perspectives. *BMJ Glob. Health* 7, e008268 (2022).

```
%%%%%%%%%%%%%%%%%%%%%%%%%%%%%%%%
%%% Code for use with Matlab %%%
%%%%%%%%%%%%%%%%%%%%%%%%%%%%%%%%


%%%%%%%%%%%%%%%%%%%%%
%%% Figure 3 code %%%
%%%%%%%%%%%%%%%%%%%%%

%%% To reproduce exact results from the paper, set the random seed:
%%% (Comment out to generate new results)
rng('default')

%%% Parameters relevant to beliefs:
wr = .9; wd = .5; % weight researchers, decision makers put on
evidence
prior = .5; % prior mean for both agents, both types of evidence
s_sq = .1; % used to calculate the prior variances

%%% Parameters relevant to evidence:
mux = .7; muy = .2; % mean of evidence stream x, evidence stream y
sigma = .25; % sigma of both types of evidence
sigma_sq = sigma^2;

%%% Other parameters:
N = 1000; % number of pieces of evidence
reps = 100; % number of times the simulation is replicated

%%% Track the policy support beliefs for the researcher, decision
maker:
%%% (First entry is just the starting beliefs)
p = zeros(2,N+1,reps);
p(:,1,:) = prior;


%%% Run the simulation:
for r=1:reps
    %%% Each replication, start over the evidence gathering:
    barx = 0; bary = 0;
for n=1:N
    %%% Each timestep, gather evidence then calculate the average of
evidence
    %%% gathered so far:
    %%% (This is done each timestep in order to track beliefs over
time.)
    evx = normrnd(mux,sigma); barx = barx/(n/(n-1)) + evx/n;
    evy = normrnd(muy,sigma); bary = bary/(n/(n-1)) + evy/n;

    %%% Agents update beliefs about the evidence:
    erx = (n*s_sq*barx + wr*sigma_sq*prior) / (n*s_sq +
wr*sigma_sq);
    ery = (n*s_sq*bary + (1-wr)*sigma_sq*prior) / (n*s_sq + (1-
wr)*sigma_sq);
```

```
    edx = (n*s_sq*barx + wd*sigma_sq*prior) / (n*s_sq +
wd*sigma_sq);
    edy = (n*s_sq*bary + (1-wd)*sigma_sq*prior) / (n*s_sq + (1-
wd)*sigma_sq);

    %%% Agents update beliefs about policy support:
    p(1,n+1,r) = wr * erx + (1 - wr) * ery;
    p(2,n+1,r) = wd * edx + (1 - wd) * edy;
end
end

%%% Calculate policy and support disagreement over time, averaged
across
%%% replications:
%%% (Per time period, sum disagreement for all reps, then divide by
# reps.)
policy = zeros(1,N+1); support = zeros(1,N+1); % create trackers
for n = 1:N+1 % per time period
    for r = 1:reps
        if (p(1,n,r) > .5 && p(2,n,r) < .5) || (p(1,n,r) < .5 &&
p(2,n,r) > .5)
            policy(1,n) = policy (1,n) + 1;
        end
        support(1,n) = support(1,n) + abs(p(1,n,r) - p(2,n,r));
    end
end
policy = policy/reps;  support = support/reps;

%%% Plot policy support beliefs over time, with two example paired
%%% trajectories:
%%% (These runs were chosen to illustrate certain possible
trajectories.)
run = 6; run2 = 7;
figure('Renderer', 'painters', 'Position', [100 210 320 250])
h = axes;
set(h,'xscale','log')
hold on
for r = 1:reps
    plot(1:N+1,p(1,:,r), "Color", [1 .7 .7])
end
for r =1:reps
    plot(1:N+1,p(2,:,r), "Color", [.7 .7 1])
end
plot(1:N+1, p(1,:,run), "Color", [.8 0 0],'LineWidth',1.5)
plot(1:N+1, p(2,:,run), "Color", [0 0 .8], 'LineWidth',1.5)
plot(1:N+1, p(1,:,run2),":", "Color", [.8 0 0],'LineWidth',2)
plot(1:N+1, p(2,:,run2),":", "Color", [0 0 .8], 'LineWidth',2)
refline([0 0.5])
xlabel('Evidence Accumulated')
ylabel('Policy Support')
x = [0.74 0.74];    % for arrow length/location
y = [0.39 0.49];      % for arrow height/width
annotation('textarrow',x,y,'HeadStyle', "deltoid", 'HeadWidth',8,
'String',' \mu_D ','FontSize',9,'Linewidth',1)
```

```
x = [0.74 0.74];
y = [0.8 0.7];
annotation('textarrow',x,y,'HeadStyle', "deltoid", 'HeadWidth',8,
'String',' \mu_R ','FontSize',9,'Linewidth',1)
ylim([0 1])
xticks([2 11 101 1001]) % because first tick no evidence is
collected
xticklabels([{'10^{0}'},{'10^{1}'},{'10^{2}'},{'10^{3}'}])

%%% Plot support disagreement over time:
figure('Renderer', 'painters', 'Position', [455 210 160 100])
h = axes;
set(h,'xscale','log')
hold on
plot(1:N+1,support, "--+",'MarkerSize',4, "Color", [.9 .5 0])
xlabel('Evidence Accumulated')
ylabel({'Support';'Disagreement'})
xticks([2 101])
xticklabels([{'10^{0}'},{'10^{2}'}])

%%% Plot policy disagreement over time:
figure('Renderer', 'painters', 'Position', [455 360 160 100])
h = axes;
set(h,'xscale','log')
hold on
plot(1:N+1,policy, "--o",'MarkerSize',2.5, "Color", [.9 .5 0])
xlabel('Evidence Accumulated')
ylabel({'Policy';'Disagreement'})
xticks([2 101])
xticklabels([{'10^{0}'},{'10^{2}'}])



%%%%%%%%%%%%%%%%%%%%%%
%%% Figure S1 code %%%
%%%%%%%%%%%%%%%%%%%%%%

%%% To reproduce exact results from the paper, set the random seed:
%%% (Comment out to generate new results)
rng('default')

%%% Parameters relevant to beliefs:
ws = .5; % weight agent puts on slower evidence stream
prior = .5; % prior mean for agent, both types of evidence
s_sq = .1; % used to calculate the prior variances
inertia = .9; % how much last round's beliefs affect this round's
beliefs

%%% Parameters relevant to evidence:
mus = .8; muf = .3; % mean of slower, faster evidence
sigma = .25; %sigma of both types of evidence
sigma_sq = sigma^2;
speed = 10; % how much faster f evidence stream is compared to s
```

```
%%% Other parameters:
N = 1000; % number of pieces of evidence
reps = 100; % number of times the simulation is replicated

%%% Track the policy support beliefs for three situations:
    %%% 1. both evidence streams are the same speed
    %%% 2. f evidence stream is faster, no inertia in beliefs
    %%% 3. f evidence stream is faster, with intertia in beliefs
%%% (First entry is just the starting beliefs)
p = zeros(3,N+1,reps);
p(:,1,:) = prior;

%%% Run the simulation:
for r=1:reps
    %%% Each replication, start over the evidence gathering:
    bars = 0; barf = 0; bars_slow = 0;
    evidence=zeros(2,N);
for n=1:N
    %%% This time, we create the evidence vectors upfront, so that
agents can
    %%% draw from them at different speeds in the different
scenarios:
    evidence(1,n) = normrnd(mus,sigma);
    evidence(2,n) = normrnd(muf,sigma);
end
for n=1:N
    %%% Agents gather evidence:
    if rem(n,speed) == 0
        bars_slow = sum(evidence(1,1:n/speed))/(n/speed);
    end
    bars = sum(evidence(1,1:n))/n;
    barf = sum(evidence(2,1:n))/n;

    %%% Agents update beliefs about evidence:

    ef = (n*s_sq*barf + (1-ws)*sigma_sq*prior) / (n*s_sq + (1-
ws)*sigma_sq);
    es = (n*s_sq*bars + ws*sigma_sq*prior) / (n*s_sq + ws*sigma_sq);
    eslow = (floor(n/speed)*s_sq*bars_slow + ws*sigma_sq*prior) /
(floor(n/speed)*s_sq + ws*sigma_sq);

    %%% Agents update beliefs about policy support:
    p(1,n+1,r) = ws * es + (1 - ws) * ef;
    p(2,n+1,r) = ws * eslow + (1 - ws) * ef;
    p(3,n+1,r) = inertia * sum(p(3,1:n,r))/n + (1-inertia)*(ws *
eslow + (1 - ws) * ef);
end
end

%%% Plot policy support beliefs over time, with an example of three
paired
%%% trajectories:
%%% (This run was chosen to illustrate a possible set of
trajectories.)
```

```
run = 20;
figure('Renderer', 'painters', 'Position', [100 210 320 250])
h = axes;
set(h,'xscale','log')
hold on
%%% We plot the three example trajectory lines first. (These get
drawn over
%%% and then redrawn on top, but this sets the line colors for the
legend.)
plot(1:N+1, p(1,:,run), "Color", [0 0 .6], 'LineWidth',1.5)
plot(1:N+1, p(2,:,run), "Color", [0 .6 0],'LineWidth',1.5)
plot(1:N+1, p(3,:,run), "Color", [.3 0 .3], 'LineWidth',1.5)
for r = 1:reps
    plot(1:N+1,p(1,:,r), "Color", [.7 .7 1])
end
for r =1:reps
    plot(1:N+1,p(2,:,r), "Color", [.7 .9 .7])
end
for r =1:reps
    plot(1:N+1,p(3,:,r), "Color", [.8 .7 .9])
end
plot(1:N+1, p(1,:,run), "Color", [0 0 .6], 'LineWidth',1.5)
plot(1:N+1, p(2,:,run), "Color", [0 .6 0],'LineWidth',1.5)
plot(1:N+1, p(3,:,run), "Color", [.4 0 .4], 'LineWidth',1.5)
refline([0 0.5])
xlabel('Evidence Accumulated')
ylabel('Policy Support')
ylim([0 1])
legend('Equal', 'Unequal','Unequal & Inertia', 'FontSize',7,
'Location', 'southeast')
legend boxoff
xticks([2 11 101 1001]) % because first tick is no evidence
collected
xticklabels([{'10^{0}'},{'10^{1}'},{'10^{2}'},{'10^{3}'}])
```
